# HIV-2 infects resting CD4+ T cells but not monocyte-derived dendritic cells

**DOI:** 10.1186/s12977-014-0131-7

**Published:** 2015-01-13

**Authors:** Lise Chauveau, Isabel Puigdomenech, Diana Ayinde, Ferdinand Roesch, Françoise Porrot, Daniela Bruni, Benoit Visseaux, Diane Descamps, Olivier Schwartz

**Affiliations:** Institut Pasteur, URA CNRS 3015, Virus & Immunity Unit, Paris, France; Université Paris Diderot, Sorbonne Paris Cité, Cellule Pasteur, Paris, 75015 France; Institut Pasteur, Hepacivirus & Innate Immunity Unit, Paris, France; IAME, UMR 1137, Université Paris Diderot, Sorbonne Paris Cité, and INSERM, Paris, 75018 France; AP-HP, Hôpital Bichat, Laboratoire de Virologie, Paris, France; Vaccine Research Institute, Hôpital Henri Mondor, Créteil, France

**Keywords:** HIV-2, Vpx, SAMHD1, Monocyte-derived dendritic cells, CD4+ lymphocytes, Interferon

## Abstract

**Background:**

Human Immunodeficiency Virus-type 2 (HIV-2) encodes Vpx that degrades SAMHD1, a cellular restriction factor active in non-dividing cells. HIV-2 replicates in lymphocytes but the susceptibility of monocyte-derived dendritic cells (MDDCs) to in vitro infection remains partly characterized.

**Results:**

Here, we investigated HIV-2 replication in primary CD4+ T lymphocytes, both activated and non-activated, as well as in MDDCs. We focused on the requirement of Vpx for productive HIV-2 infection, using the reference HIV-2 ROD strain, the proviral clone GL-AN, as well as two primary HIV-2 isolates. All HIV-2 strains tested replicated in activated CD4+ T cells. Unstimulated CD4+ T cells were not productively infected by HIV-2, but viral replication was triggered upon lymphocyte activation in a Vpx-dependent manner. In contrast, MDDCs were poorly infected when exposed to HIV-2. HIV-2 particles did not potently fuse with MDDCs and did not lead to efficient viral DNA synthesis, even in the presence of Vpx. Moreover, the HIV-2 strains tested were not efficiently sensed by MDDCs, as evidenced by a lack of MxA induction upon viral exposure. Virion pseudotyping with VSV-G rescued fusion, productive infection and HIV-2 sensing by MDDCs.

**Conclusion:**

Vpx allows the non-productive infection of resting CD4+ T cells, but does not confer HIV-2 with the ability to efficiently infect MDDCs. In these cells, an entry defect prevents viral fusion and reverse transcription independently of SAMHD1. We propose that HIV-2, like HIV-1, does not productively infect MDDCs, possibly to avoid triggering an immune response mediated by these cells.

**Electronic supplementary material:**

The online version of this article (doi:10.1186/s12977-014-0131-7) contains supplementary material, which is available to authorized users.

## Background

HIV-1 and HIV-2 share many similarities in their genetic organization, modes of replication and potential interaction with their hosts [1]. However, major differences exist regarding the clinical consequences of infection. In the absence of antiretroviral treatment, progression to immunodeficiency is more rare and occurs more slowly with HIV-2 than with HIV-1 [1,2]. About 10-30% of HIV-2-infected patients are long-term non-progessors or viral controllers [3-5]. Viral RNA levels in HIV-2 infection are about 30 times lower than in HIV-1 infection, and viral DNA levels are also reduced [1,2,6-9]. However, as for HIV-1, disease progression in HIV-2-infected individuals has been associated with increased chronic immune activation, CD4+ T cell depletion and alterations in circulating monocytes and myeloid DCs [1,2,10-13].

Why HIV-2 is less pathogenic than HIV-1, and why HIV-2 provides partial cross-protection against HIV-1 disease progression in dually infected individuals, remains partly understood [1,4,14,15]. It has been proposed that HIV-2 replication may be more efficiently controlled by the immune response. For instance, most of HIV-2-infected individuals produce neutralizing antibodies [2,16]. HIV-2 is more sensitive to neutralization than HIV-1, likely because HIV-2 Env proteins expose multiple cross-reactive epitopes and have fewer glycosylation sites in the V3 loop than HIV-1 [17-19]. In addition to a strong humoral response, HIV-2-infected donors have well-preserved and polyfunctional HIV-specific CD4+ and CD8+ T cell responses that are associated with delayed disease progression [20-22]. The innate immune response may also be very active against HIV-2. It has been reported that *in vitro*, HIV-1 is more resistant to type-I IFN than HIV-2 and SIVmac [23]. This suggests that some cellular restriction factors, induced by type-I IFN, may be particularly active against HIV-2. For example, HIV-2 is more sensitive to restriction by Trim5α than is HIV-1 [24]. There are strain-specific variations in HIV-2 sensitivity to Trim5α, depending on motifs within the capsid [25]. Lv2 is another example of an unidentified factor that restricts infection of some HIV-2 viruses, but not HIV-1, following virus entry [26,27]. The block manifests after reverse transcription but prior to nuclear entry, and sensitivity to restriction maps to the HIV-2 envelope and capsid genes [26,27]. Tetherin, a cellular protein blocking viral release, is also differently counteracted by HIV-1 and HIV-2. In HIV-1, the anti-tetherin activity is conferred by Vpu, whereas in HIV-2, the intracytoplasmic portion of Env mediates this effect [28,29]. Another protein, the recently identified RNA-associated early-stage anti-viral factor (REAF), inhibits both HIV-1 and HIV-2 just after cell entry [30].

The restriction factor SAMHD1 blocks HIV replication by degrading intracellular dNTPs and HIV-1 RNA [31-37]. SAMHD1 inhibits reverse transcription in non-diving cells, such as monocytes, macrophages, dendritic cells and non-activated CD4+ lymphocytes [31-36]. In dividing cells such as activated CD4+ T cells, SAMHD1 is phosphorylated and does not restrict HIV-1 [38-40]. HIV-2 and some SIV strains encode the accessory protein Vpx, which degrades SAMHD1 and allows escape from this restriction, whereas HIV-1 lacks a Vpx-like activity. The anti-SAMHD1 activity is conserved in Vpx alleles isolated from viremic or aviremic HIV-2-infected individuals [41]. It has been proposed that HIV-2, through the SAMHD1-degrading action of Vpx, may trigger a more efficient immune response by productively infecting dendritic cells (DCs) [42]. HIV-1, in sparing SAMHD1, may avoid productive infection of DCs and thus limit the resulting protective type-I IFN response mounted by these cells [35,43-45]. In addition to degrading SAMHD1, Vpx may also inhibit the function of IRF-5 [46]. Of note, HIV-1 and HIV-2 differentially interact with other target cells. The kinetics of HIV-1 and HIV-2 replication are different in human primary macrophage cultures [47]. Exposure of plasmacytoid DCs (pDCs) to HIV-1 and HIV-2 differentially mature the cells into IFN-producing cells or Antigen Presenting Cells [48].

The exact role of Vpx during HIV-2 replication is not fully understood. A comparison between wild-type and Vpx-deleted HIV-2 demonstrated that Vpx is not necessary for viral replication in activated CD4+ T cells and in T cell lines [49-51]. In contrast, HIV-2 infection is poorly supported in resting (non-stimulated) CD4+ T cells. Even in the presence of Vpx, less than 2% of non-stimulated CD4+ T cells are productively infected [36]. Vpx is also required for the productive infection of macrophages by HIV-2 virions pseudotyped with VSV-G [41]. There is controversy regarding the sensitivity of DCs to HIV-2 infection. It has been reported that HIV-2 efficiently replicated in monocyte-derived DCs (MDDCs), using the laboratory-adapted HIV-2 ROD strain, pseudotyped with VSV-G [42,44]. This efficient replication was associated with an immune detection of incoming viral cDNA by the innate sensor cGAS [42] and with DC maturation. Others reported that neither CCR5-tropic primary HIV-2 isolates nor a CXCR4-tropic laboratory-adapted viral strain could efficiently infect primary myeloid DCs or pDCs, though these viruses could infect primary CD4+ T cells [52]. Moreover, HIV-2 exposure did not promote full maturation of DCs [41,52]. These different results may be due to different experimental systems, DC subsets, or viral strains, and/or to the use of VSV-G-pseudotyped HIV-2 in some studies.

In this study, we explored some virological and immunological aspects of the interaction of HIV-2 with CD4+ T cells and MDDCs. As previously described, HIV-2 spread in primary activated lymphocytes. In resting T cells, productive HIV-2 infection was not detected, but subsequent activation of HIV-2-exposed lymphocytes led to viral replication in a Vpx-dependent manner. In contrast, monocyte-derived DCs (MDDCs) were poorly sensitive to HIV-2 infection, even in the presence of Vpx, and the HIV-2 isolates tested did not trigger a strong type-I IFN response in MDDCs. Therefore, our data do not support the hypothesis that Vpx-mediated SAMHD1 degradation allows HIV-2 productive infection of MDDCs, and thus a potential difference in the ability of HIV-1 and HIV-2 to infect this cell type may not underlie differences in disease pathogenesis.

## Results

### HIV-2 primary strains replicate in activated CD4+ T cells and infect resting CD4+ T cells

**Figure 1:**
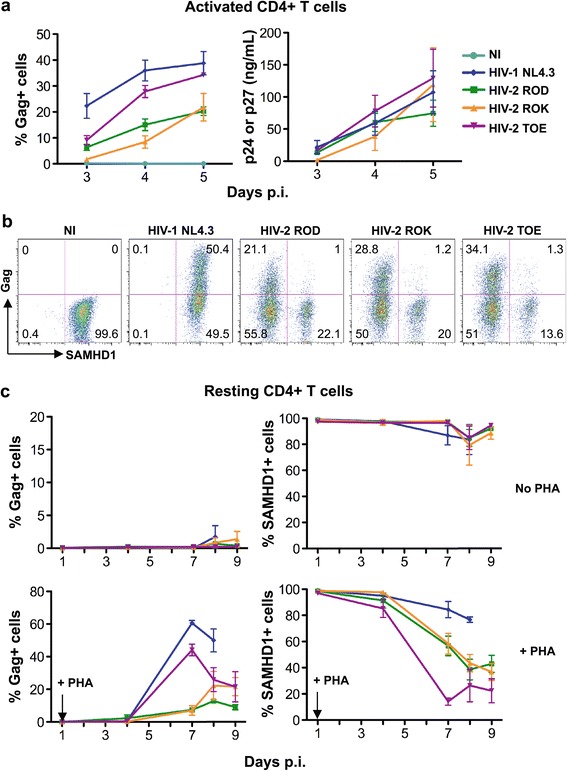
**HIV-2 replication in activated and in unstimulated CD4+ T cells. (a)** Replication of different HIV-2 isolates in activated CD4+ T cells. Primary activated CD4+ T cells were infected with the laboratory-adapted HIV-2 strain ROD or with TOE and ROK, two HIV-2 isolated from infected patients (viral inoculum was 30 ng p27 mL^−1^). The HIV-1 NL strain (30 ng p24 mL^−1^) was used as a control. Viral replication was followed by intracellular Gag staining and quantification of Gag release in the supernatants. NI: Non infected. Results are Mean ± SEM of 6 independent donors. **(b)** SAMHD1 down-regulation. At day 5 post-infection (p.i.), Gag and SAMHD1 levels were analyzed by flow cytometry. One representative donor, out of 6, is shown. **(c)** Infection of resting, unstimulated CD4+ T cells with different HIV-2 strains. Unstimulated CD4+ T cells were exposed to HIV-2 ROD, ROK (30 ng p27 mL^−1^) or TOE (15 ng p27 mL^−1^) for 4 h. One day post-infection, cells were activated or not with PHA/IL-2. Gag and SAMHD1 levels were measured over time by flow-cytometry. Data are Mean ± SEM of 5 independent donors.

We first verified that the different HIV-2 strains used in this study replicated in primary CD4+ T cells. We purified CD4+ T cells from PBMCs from healthy donors, exposed them to phytohemaglutinin (PHA) to induce activation, and incubated them with viral particles normalized for p27 antigen levels. We used two primary viral biological isolates, a group B dual-tropic strain (ROK) and a group A X4-tropic strain (TOE), both isolated from aviremic patients (see materials and methods for a description of the viruses), and as a reference the group A dual-tropic prototype ROD isolate. Viral replication was assessed by flow cytometry, after intracellular staining with the anti-HIV-1 Gag monoclonal antibody KC57, which also recognizes HIV-2 Gag [52]. The HIV-2 isolates replicated in CD4+ T cells, with 15-30% of Gag + cells at day 5 post-infection (p.i.) (Figure 1a). HIV-1 (NL4.3 strain) efficiently replicated in the same cells (Figure 1a). The appearance of HIV-2 Gag + cells was associated with viral release in the supernatants, reaching 50–100 ng mL^−1^ of Gag p27 at day 5 p.i. (Figure 1a). HIV-2 encodes Vpx, which degrades the restriction factor SAMHD1 [31-36]. As expected, we observed a strong decrease of SAMHD1 in HIV-2-infected (Gag+) cells whereas HIV-1 replicated without degrading SAMHD1 (Figure 1b). With HIV-2, the SAMHD1-negative and Gag-negative population (Figure 1b) may represent cells in which incoming Vpx degraded SAMHD1 before, or in the absence of, productive infection.

We then asked whether HIV-2 infects resting CD4+ lymphocytes. We purified unstimulated CD4+ T cells from PBMCs, and verified that >95% did not express the activation and proliferation markers CD69 and Ki67 (Additional file 1: Figure S1). These cells were exposed to the HIV-2 isolates ROD, ROK or TOE as well as to HIV-1 (NL4.3) and were cultivated with minimal doses of IL-2 to prevent cell death. As expected [36], no viral replication was detected in these cells up to 9 days post viral challenge (Figure 1c). Of note, the slight down-regulation of SAMHD1 observed in non-activated CD4 + T cells (in about 10% of the cells) over time (mostly after 7–9 days of culture) may represent dead or dying cells (Figure 1c). However, PHA activation, one day following viral exposure, led to significant HIV-1 and HIV-2 replication. In the case of HIV-2, concomitant and strong SAMHD1 down-regulation was observed in activated cells (Figure 1c). Similar results were obtained when CD4+ T cells were activated 4 days after viral exposure (not shown).

### Role of HIV-2 Vpx in primary CD4+ T cells

**Figure 2:**
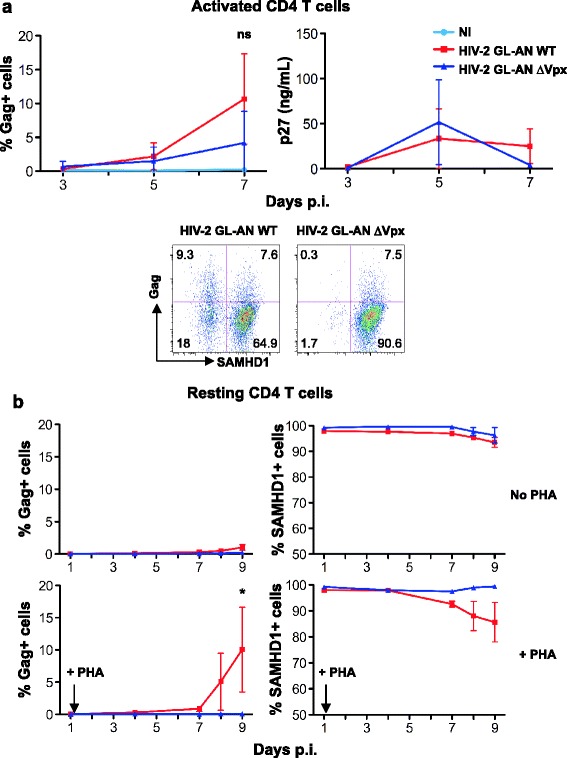
**Vpx is necessary for HIV-2 infection in unstimulated CD4+ T cells. (a)** Replication of HIV-2 GL-AN, expressing or not Vpx, in activated CD4+ T cells. Primary activated CD4+ T cells were infected with HIV-2 GL-AN WT and GL-AN ∆Vpx (20–30 ng p27 mL^−1^). Viral replication was followed as in Figure 1a. Upper panels: Mean ± SD of 6 independent donors. Lower panels: Gag and SAMHD1 expression at day 7 p.i., in one representative donor. ns: non significant **(b)** Role of Vpx in HIV-2 infection of resting, unstimulated CD4+ T cells. Unstimulated CD4+ T cells were exposed to HIV-2 GL-AN WT and GL-AN ∆Vpx (30 and 90 ng p27 mL^−1^, respectively) as described in Figure 1c. One day post-infection, the cells were activated with PHA/IL-2. Gag and SAMHD1 levels are shown at different days p.i. Data are Mean ± SEM of 3 independent donors. *: p-value < 0.05.

To assess the role of Vpx in infection of activated or unstimulated CD4+ T cells, we followed replication of an HIV-2 molecular clone, the GL-AN provirus, expressing or not the viral accessory protein (GL-AN WT and ∆Vpx, respectively) [50]. GL-AN is a chimerical strain, originating from a virus isolated in a Ghanaian patient, and containing a large part of the HIV-2 ROD *env* sequence [50]. GL-AN replicated in activated primary CD4+ T cells, and the absence of Vpx was associated with a slight but non-significant decrease of viral growth (Figure 2a). GL-AN WT, and not ∆Vpx down-regulated SAMHD1 in infected cells (Figure 2a). With GL-AN WT, the extent of SAMHD1 down-regulation was less marked than with ROD, ROK and TOE (Figure 1b). This suggests that, as previously reported [41], Vpx from different viral isolates down-regulate SAMHD1 with various efficiencies. However, with GL-AN WT, the majority of Gag + cells were SAMHD1-negative, whereas ∆Vpx replicated in SAMHD1-positive cells (Figure 2a). This is not surprising since SAMHD1 is phosphorylated in cycling cells, and this phosphorylation inactivates the enzyme [38-40].

In resting CD4+ T cells, as observed with the other HIV-2 strains, the GL-AN viruses did not lead to productive infection. However, GL-AN WT, but not GL-AN ∆Vpx, replicated in CD4+ T cells stimulated one day (Figure 2b) or four days (not shown) following viral challenge. This replication was associated with a down-regulation of SAMHD1.

Altogether, these results suggest that upon HIV-2 exposure, unstimulated lymphocytes harbor virus but do not produce viral antigens. This may be due to pre-integration defects and/or a HIV LTR transcription block associated with their quiescent status [31]. Moreover, this non-productive infection requires Vpx, and leads to viral outgrowth after lymphocyte stimulation.

### Monocyte-derived dendritic cells are poorly sensitive to HIV-2

**Figure 3:**
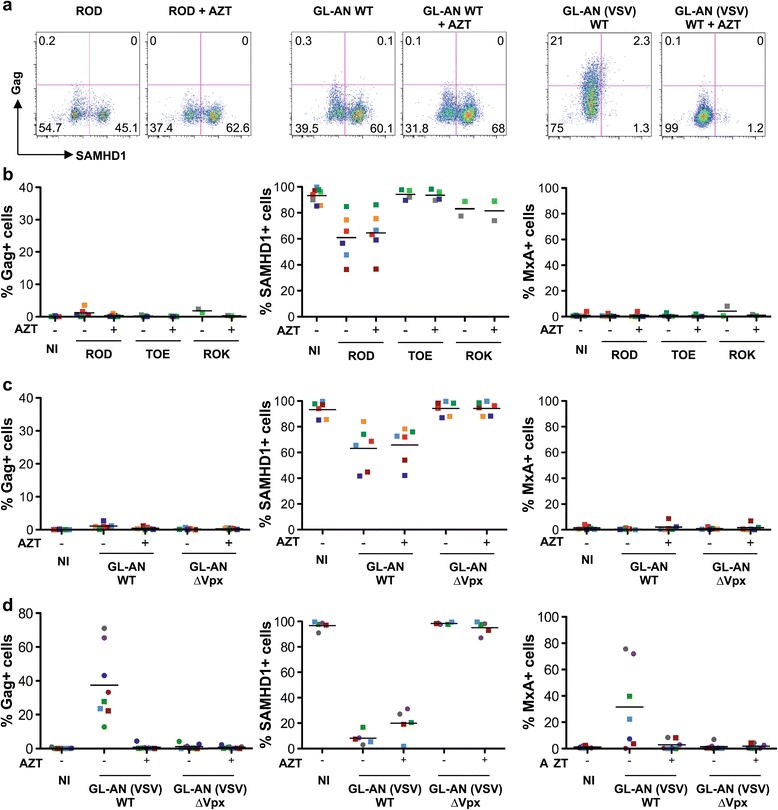
**HIV-2 infection of monocyte-derived dendritic cells (MDDCs). (a)** Infection of MDDCs by primary HIV-2. MDDCs were exposed to HIV-2 ROD, GL-AN (150 ng p27 mL-1) and GL-AN (VSV) (50 ng p27 mL-1). After 3 days, the levels of Gag and SAMHD1 were measured by flow cytometry. A representative experiment is depicted. **(b)** Infection with HIV-2 ROD, TOE (150 ng p27 mL-1) and ROK (100 ng p27 mL-1). After 3 days, the levels of Gag, SAMHD1 and MxA were measured by flow cytometry. Data are Mean of 6 independent donors. The innate response was analyzed by measuring by flow cytometry the levels of the interferon-stimulated gene MxA. **(c**) Infection of MDDCs by HIV-2 GL-AN expressing or not Vpx. MDDCs were exposed to HIV-2 GL-AN WT and GL-AN ∆Vpx (150 ng p27 mL-1) and analyzed as described in (b). **(d)** Infection of MDDCs with HIV-2 GL-AN WT or ∆Vpx virions pseudotyped with VSV-G. MDDCs were exposed to HIV-2 GL-AN (VSV) WT and GL-AN (VSV) ΔVpx (50 ng p27 mL-1) and analyzed as described in **(b)**.

We then asked whether MDDCs may be infected by HIV-2 and evaluated the consequences of viral exposure on expression of the interferon-stimulated gene MxA [53]. Cells were exposed to the HIV-2 strains ROD, TOE, ROK, and to GL-AN WT or ∆Vpx. The extent of infection was measured by double staining for Gag and SAMHD1 after 3 days and analyzing by flow cytometry (Figure 3a). One representative experiment is shown in Figure 3a, and the values obtained from up to 6 independent donors are shown in Figure 3b,c. At the high dose of virus used (150 ng p27 mL^−1^), very few Gag + cells were detected (<3% of MDDCs were Gag + at 72 h, Figure 3b) and this percentage did not further increase at 96 h p.i. (Additional file 1: Figure S1c). Augmenting the viral inoculum to 500 ng p27 mL^−1^ did not improve the efficiency of infection, nor did pre-treatment of the cells with SIV-derived virus like particles carrying Vpx that completely down-regulated SAMHD1 (not shown). SAMHD1 was partly down-regulated in cells exposed to ROD and, to even a lesser extent, to ROK (which are dual-tropic viruses), but this was not observed for the X4-tropic TOE strain (Figure 3b). GL-AN WT, and not ∆Vpx, also partly down-regulated SAMHD1 (Figure 3c). This suggests that incoming Vpx present in the virions could access the cytoplasm of target cells to a certain extent. This low efficiency of productive infection with ROD, TOE, ROK and GL-AN isolates did not lead to the induction of MxA (Figure 3 b,c).

### VSV-G pseudotyping allows productive HIV-2 infection and viral sensing in MDDCs

The fact that the various HIV-2 strains tested promoted only a partial down-regulation of SAMHD1 suggested that viral entry was impaired in MDDCs. We thus examined the effect of VSV-G pseudotyping on HIV-2 infection by exposing MDDCs to GL-AN (VSV) WT and GL-AN (VSV) ∆Vpx virions. One representative experiment is shown in Figure 3a, and the values obtained with 8 independent donors are shown in Figure 3b,c. Depending on the donor, we observed 10-70% of Gag + cells following exposure to GL-AN (VSV) WT. The Gag signal corresponded to newly synthesized viral proteins, since it was no longer detected in the presence of the reverse transcriptase inhibitor AZT (Figure 3d). This efficient infection was associated with a potent down-regulation of SAMHD1 in up to 90% of the cells. SAMHD1 remained down-regulated in the presence of AZT, indicating that incoming Vpx was responsible for this effect. Moreover, GL-AN (VSV) WT triggered expression of MxA (Figure 3d and Additional file 1: Figure S1b). AZT inhibited MxA production, indicating that viral DNA synthesis was required to mediate sensing of GL-AN (VSV). We then examined the role of Vpx. GL-AN (VSV) ∆Vpx did not productively infect MDDCs nor did it down-regulate SAMHD1 (Figure 3d). Moreover, this virus did not induce MxA in target cells.

Taken together, these results show that VSV-G pseudotyping allows efficient infection of MDDCs by HIV-2. This productive infection leads to sensing of the virus.

### VSV-G pseudotyping increases HIV-2 fusion and reverse transcription in MDDCs

**Figure 4:**
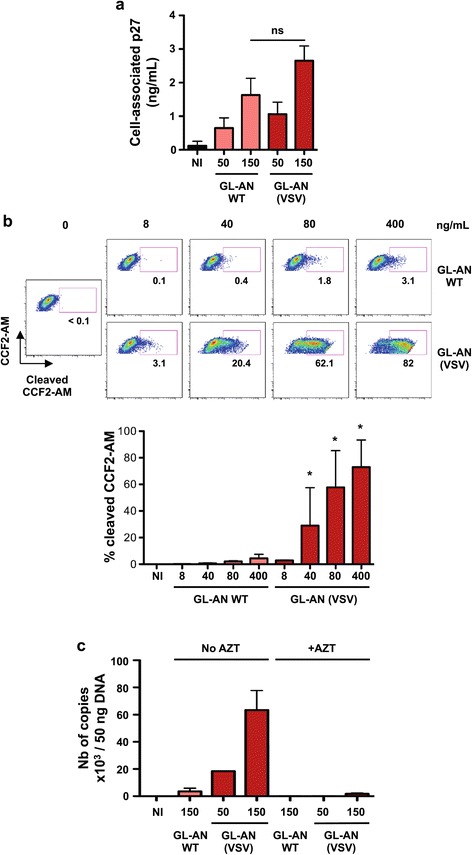
**VSV-G pseudotyping allows HIV-2 entry in MDDCs. (a)** HIV-2 binding. MDDCs were exposed to the indicated doses of HIV-2 GL-AN, pseudotyped or not with VSV-G. After 2 h at 4°C, cells were extensively washed and the amount of cell-associated p27 was assessed by ELISA. Data are Mean ± SD of 4 independent donors. ns: non significant **(b)** HIV-2 fusion. MDDCs were exposed to the indicated doses of HIV-2 GL-AN, pseudotyped or not with VSV-G, and bearing the chimeric protein β-lactamase-Vpr. After 2 h at 37°C, viral access to the cytoplasm was assessed by flow cytometry, using the ability of β -lactamase to cleave the cytoplasmic CCF2-AM fluorogenic substrate. One representative donor is shown in the upper panel and a mean ± SD of 3 independent donors in the lower panel. *: p-value < 0.05. Comparisons were made between the condition indicated and the no VSV condition at the same viral inoculum. **(c)** HIV-2 DNA synthesis. MDDCs were exposed to HIV-2 GL-AN, pseudotyped or not with VSV-G, in the presence or absence of AZT. After 3 days, the cells were harvested for HIV-2 DNA quantification by qPCR. Data are mean ± SD of 2 independent donors.

**Figure 5:**
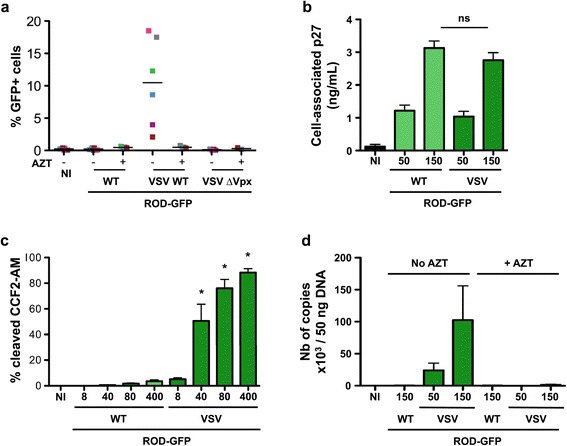
**HIV-2 ROD-GFP and MDDCs. (a)** Susceptibility of MDDCs to HIV-2 ROD-GFP infection. MDDCs were exposed to HIV-2 ROD-GFP, pseudotyped or not with VSV-G (50 and 150 ng p27 mL-1, respectively). After 3 days, the levels of GFP were measured by flow cytometry. Results from 6 independent donors are shown **(b)** HIV-2 ROD-GFP binding. MDDCs were exposed to the indicated doses of HIV-2 ROD-GFP, pseudotyped or not with VSV-G. After 2 h at 4°C, cells were extensively washed and the amount of cell-associated p27 was assessed by ELISA. Data are Mean ± SEM of 4 independent donors. ns: non significant **(c)** HIV-2 ROD-GFP fusion. MDDCs were exposed to the indicated doses of HIV-2 ROD-GFP, pseudotyped or not with VSV-G, and bearing the chimeric protein β-lactamase-Vpr. After 2 h at 37°C and 2 h at room temperature, viral access to the cytoplasm was assessed by flow cytometry, using the ability of β -lactamase to cleave the cytoplasmic CCF2-AM fluorogenic substrate. A mean ± SEM of 4 independent donors is shown. *: p-value < 0.05. Comparisons were made between the condition indicated and the no VSV condition at the same viral inoculum. **(d)** HIV-2 ROD-GFP DNA synthesis. MDDCs were exposed to HIV-2 ROD-GFP pseudotyped or not with VSV-G, in the presence or absence of AZT. After 3 days, the cells were harvested for HIV-2 DNA quantification by qPCR. Data are mean ± SEM of 4 independent donors.

We characterized the replicative defect of HIV-2 in MDDCs by comparing the ability of wild-type and VSV-G-pseudotyped virus to bind cells, undergo fusion and perform reverse transcription. To measure viral binding at the cell surface, we incubated MDDCs with increasing doses of GL-AN and GL-AN (VSV) (50 and 150 ng p27 mL^−1^) for 2 h at 4°C. After extensive washes, p27 Gag levels were measured in cell lysates by ELISA. The viruses bound to the cells in a dose dependent manner (Figure 4a). VSV-G pseudotyping resulted in a slight but non-significant increase in viral binding (Figure 4a). We then performed a viral fusion assay to assess the post-binding step of the viral cycle. HIV-2 Vpr, which is incorporated into viral particles, was fused with ß-lactamase (Blam-Vpr2) [54,55]. The successful cytoplasmic access of Blam-Vpr2, as a result of viral fusion, after 2 h of infection, was monitored by enzymatic cleavage of CCF2-AM, a fluorogenic substrate of ß-lactamase loaded in target cells [54,55]. A representative experiment is shown in Figure 5b. A dose–response analysis of the viral inoculum (8 to 400 ng p27 mL^−1^) indicated that wild-type HIV-2 fusion in MDDCs was low (Figure 4b). In sharp contrast, a positive fusion signal was detected with GL-AN (VSV), starting at the lowest viral inoculum tested (Figure 4b). A side-by-side comparison indicated that fusion of the VSV-G-pseudotyped HIV-2 with MDDCs was 50 times more efficient than that of the wild-type virus. We then quantified the reverse transcription products at day 3 post-infection. In line with the results obtained in the fusion assay, GL-AN did not permit an efficient synthesis of viral DNA, even at a high viral inoculum. In contrast, infection with VSV-G-pseudotyped HIV-2 was associated with high levels of viral DNA. This signal corresponded to newly synthesized molecules since it was inhibited by AZT (Figure 4c).

Similar results were obtained when using another HIV-2 strain (ROD-GFP) pseudotyped or not with VSV-G. ROD-GFP is derived from ROD9 and carries the GFP gene in place of Nef [36]. We therefore used the GFP signal to quantify productively infected cells. ROD-GFP did not lead to productive infection of MDDCs, whereas VSV-G pseudotyping rescued viral infectivity (Figure 5a). ROD-GFP bound to MDDCs (Figure 5b), but was unable to fuse (Figure 5c) and to promote viral DNA synthesis (Figure 5d).

Altogether, these results indicate that HIV-2 poorly infects MDDCs because of a defect in target cell entry. This defect is overcome by VSV-G pseudotyping, which rescues viral infectivity in these cells.

## Discussion

We studied here HIV-2 replication in primary CD4+ T cells and in MDDCs. We used two primary HIV-2 strains (ROK and TOE), as well as the reference isolate ROD. We focused on the role of Vpx by using the HIV-2 molecular clones GL-AN and ROD-GFP, expressing or not this viral protein.

The HIV-2 isolates replicated in activated CD4+ T cells, as evidenced by the appearance of Gag + cells and by the release of Gag p27 in the supernatants. At each time point, the fraction of Gag + cells was lower than with the HIV-1 strain (NL4.3) tested in parallel. For instance, at day 4 p.i., about 40% of the cells were Gag + with HIV-1, whereas the different HIV-2 strains led to 5 to 25% of infected cells. It is however difficult to formally conclude that HIV-2 spreads less potently than HIV-1 in activated T cells because only one HIV-1 strain was tested here in parallel. Of note, the anti-Gag antibody used in this study (KC57) may recognize HIV-2 antigens less efficiently than those of HIV-1. However, the use of another anti-HIV-2 Gag-specific mAb (25/26A) did not lead to the detection of an increased number of infected cells (not shown). Accordingly, it has been previously reported that in PBMCs infected *in vitro*, the total amount of viral DNA is lower with HIV-2 ROD than with HIV-1 NL4.3 [56]. We further observed that the mean fluorescence intensity (MFI) of Gag staining was higher with HIV-1 than with HIV-2. This may again reflect a different affinity of the antibody, or may indicate that the number of Gag proteins per infected cell is relatively low with HIV-2.

We characterized the role of Vpx during HIV-2 replication in primary CD4+ T cells. As expected [49-51], both WT and ∆Vpx HIV-2 replicated in PHA-activated lymphocytes, with replication decreasing slightly in the absence of Vpx. Interestingly, all HIV-2 isolates led to a down-regulation of SAMHD1 in infected cells, whereas HIV-1 replicated without degrading this cellular restriction factor. Overall, Vpx was not necessary for HIV-1 or HIV-2 infection in activated CD4+ T cells. This was expected since SAMHD1 is phosphorylated and inactive in cycling cells [38-40]. However, the role of Vpx in the infection of unstimulated primary lymphocytes was dramatic. When exposed to HIV-2, these cells did not become productively infected. PHA stimulation, one or four days following viral exposure, promoted production of HIV-2 ROD, ROK, TOE and GL-AN WT, associated with SAMHD1 down-regulation, whereas GL-AN ∆Vpx did not replicate in this setting. Therefore, Vpx allows HIV-2 infection of resting CD4+ T cells, where SAMHD1 is active. Interestingly, HIV-1 also replicates in CD4+ T cells activated after viral exposure [57,58], without degrading SAMHD1. This may be due to the efficiency of the HIV-1 reverse transcriptase, which displays a low Kd for dNTPs, and may thus function in non-cycling cells with a low dNTP environment [33,34,59,60]. Therefore, both HIV-1 and HIV-2 infect non-cycling CD4+ T cells [31,36], through different mechanisms. HIV-2 may require Vpx to antagonize SAMHD1, in order to increase the intracellular levels of dNTPs or to avoid degradation of viral nucleic acids, probably because the reverse transcription process is less efficient for HIV-2 than for HIV-1 [61].

Discrepant results have been reported in the literature regarding the interaction of HIV-2 with MDDCs in cell culture systems [42,44,52]. We show here that MDDCs are poorly susceptible to HIV-2 replication, using either the primary viral isolates ROK or TOE, the laboratory-adapted strain ROD, or the molecular clones GL-AN and ROD-GFP. These results confirm and extend those of Duvall et al. [52], who showed that primary HIV-2 strains, isolated from either viremic and aviremic patients, do not detectably infect myeloid DCs. We also observed that upon exposure with HIV-2, SAMHD1 was partly down-regulated in MDDCs. This suggests that Vpx could access the cytoplasm and that viral fusion could occur to some extent. Future work will help understanding why the partial down-regulation of SAMHD1 was not associated with productive HIV-2 infection. Furthermore, we show here that the GL-AN strain, when pseudotyped with VSV-G, gained the capacity to infect MDDCs. This process required Vpx, since GL-AN (VSV) ∆Vpx did not infect these cells. Therefore, primary HIV-2 isolates do not potently infect MDDCs. That VSV-G rescued infection strongly suggests that an early stage of the viral cycle is restricted in these cells. We further determined that viral binding to MDDCs occurred normally with HIV-2. This is not surprising since HIV binding may involve multiple receptors and molecules (CD4, lectins like DC-SIGN and Siglec/CD169, heparan sulfate, etc.…) and does not necessarily lead to productive infection. However, viral fusion was severely impaired with viruses bearing the HIV-2 envelope. Accordingly, viral DNA synthesis did not occur efficiently. Both viral fusion and DNA synthesis were rescued by VSV-G pseudotyping. It will be worth determining if sensitivity to restriction factors such as LV-2 or REAF [26,30,62] are relieved when viral particles are pseudotyped with VSV-G. Our results also suggest that the productive infection of MDDCs reported in the literature [41,42,44] was due to VSV-G pseudotyping, or to the use of HIV-2 strains with a strong tropism for these cells. It will also be important to determine whether the distinct myeloid DC subsets that have been identified [63] show different susceptibility to HIV-2 replication.

The low susceptibility of MDDCs to infection by HIV-2 primary isolates was associated with a poor triggering of an innate immune response. In cells exposed to HIV-2, we did not observe induction of the interferon-stimulated gene MxA. Our results confirm those of Duvall et al., who did not observe cell maturation or cytokine production by myeloid DCs exposed to primary HIV-2 isolates or to the CBL-20 laboratory-adapted virus [52]. In addition, we report that infection with VSV-pseudotyped HIV-2 triggered MxA expression in MDDCs. It has been recently reported that the capsids of HIV-2 are determinants for immune detection of viral cDNA in MDDCs by the sensor cGAS [42]. These results were obtained with VSV-G-pseudotyped HIV-2 ROD. Further work will assess how natural HIV-2 isolates can be sensed by cGAS [64] in MDDCs and other cell types.

Altogether, our results strongly suggest that primary HIV-2 isolates are not efficiently sensed by MDDCs. This does not support the hypothesis that HIV-2, endowed with Vpx, may naturally infect these cells to trigger a protective immune response. We conclude rather that HIV-1 and HIV-2 avoid immune detection in MDDCs through different mechanisms. HIV-1 does not efficiently infect these cells because of a lack of a Vpx-like activity, whereas HIV-2 is restricted at an early entry stage that is not linked to SAMHD1 blockade.

Our findings that MDDCs are not sensitive to HIV-2 infection are in agreement with *in vivo* observations [65]. It has been reported that in Macaques, myeloid cells were not the site of productive SIV infection, irrespectively of Vpx. Rather, the viral DNA present in myeloid cells of these animals resulted from phagocytosis of infected T cells [65].

## Conclusion

Overall, our results extend previous studies on the interaction of HIV-1 and HIV-2 with CD4+ T cells and DCs. We show that various HIV-2 strains infect activated CD4+ T cells. HIV-2 also latently infects non-stimulated CD4+ T cells. The primary HIV-2 strains tested do not naturally replicate in MDDCs. The main differences between HIV-1 and HIV-2 regarding their interactions with CD4+ T cells and MDDCs include: (i) a potentially lower level of HIV-2 replication in primary activated CD4+ T cells; (ii) a distinct molecular mechanism to target non-activated CD4+ T cells (HIV-2 necessitates Vpx, whereas HIV-1 infection occurs without the need of a Vpx-like activity); (iii) a low efficiency of HIV-2 infection in MDDCs despite the presence of Vpx. It is tempting to speculate that some of these variations may improve our understanding of why the natural course of HIV-2 infection *in vivo* is different from that of HIV-1 [1,2].

## Methods

### Cells and reagents

PBMCs were isolated from the blood of healthy donors by Ficoll centrifugation. The blood was provided by the EFS (Etablissement français du sang, the Official French blood bank). CD4+ T lymphocytes were isolated from PBMCs by positive selection using magnetic beads (Miltenyi Biotech). When stated, CD4+ T cells were activated by PHA (1 μg mL^−1^) and grown with IL-2 for 3 days before infection. Primary CD4+ lymphocytes were grown in RPMI medium with 10% heat-inactivated fetal bovine serum (FBS). Monocytes were isolated using a CD14+ selection kit (Miltenyi Biotech). MDDCs were generated by culturing monocytes with 50 ng mL^−1^ IL-4 and 10 ng mL^−1^ GM-CSF for 5 days. The reverse transcriptase inhibitor AZT (25 μM) was from the NIH AIDS reagents program.

### Viruses

The ROK primary HIV-2 was isolated nine years after initial diagnosis from an aviremic stage C patient with low CD4 counts (18 and 24 CD4+ T cells/μL at diagnosis and sampling time, respectively). The TOE primary HIV-2 was isolated 3 years after initial diagnosis. During this period, the patient always presented undetectable viral load and a high CD4 cell count (404 to 436 CD4+ T cells/μL at diagnosis and sampling time, respectively). Viruses were isolated by co-cultivation of patient cells with a pool of PHA-activated PBMCs of healthy donors [66]. The primary viruses, as well as the prototype ROD HIV-2 strain isolated from a Cape Verdian patient [67] were grown either on PHA-stimulated PBMC or in HUT-78 T cells. The pGL-AN WT and ΔVpx plasmids (a kind gift from Florence Margottin-Goguet) [50] were used to produce viruses by transfection of 293 T cells as described or by amplification on HUT-78 cells. TOE is a X4-tropic virus, whereas ROD and ROK are dual tropic viruses [68]. GL-AN carries a large part of the ROD envelope and likely displays the same receptor usage as ROD. ROD-GFP (a kind gift of Oliver Keppler and Oliver Fackler) is derived from the ROD9 provirus and encodes GFP in Nef [36]. Vesicular Stomatitis Virus type G (VSV-G) pseudotyped viruses GL-AN WT and GL-AN ∆Vpx were generated by cotransfection of 293 T cells with the corresponding proviruses and a VSV-G expression plasmid. The SIV3+ WT and the ∆Vpx plasmids (a kind gift from Monsef Benkirane) were used to produce VLP as described [32,69].

### HIV-2 infection

Primary CD4+ T cells were exposed to the indicated virus (15 to 90 ng mL^−1^ of p27) for 3 h at 37°C in the presence of 2 μg mL^−1^ of DEAE-Dextran (Sigma-Aldrich). The infection was then monitored by flow cytometry analysis after intracellular Gag staining (KC-57 mAb, Coulter) or by p27 ELISA (Zeptometrix) on the culture supernatants. For infection of non-activated CD4+ T cells, the virus was extensively washed after 4 h at 37°C. Half of the cells were activated with PHA (PHA16, Oxoid, 1 μg mL^−1^) at day 1 or 4 post infection. The other half was kept in the presence of low concentration of IL-2 (10 U mL^−1^). The infection was then followed by flow cytometry at the indicated days.

MDDCs were seeded in flat-bottomed 96-well plates at 1×10^5^ cells per well. Cell-free infections in MDDCs were performed using indicated viral doses (50 ng mL^−1^ of p27 for the VSV-G pseudotyped viruses and 150 to 500 ng mL^−1^ of p27 for the non-pseudotyped viruses). AZT was added 2 h before infection and kept throughout the infection.

### Flow cytometry

For Gag and SAMHD1 staining, cells were fixed with PFA 4% for 10 min followed by permeabilization with PBS-Triton 0.5% for 20 min. Cells were stained either with the mouse monoclonal antibody (mAb) anti-SAMHD1 (clone I19-18) coupled with Alexa 488 [35] or with a rabbit polyclonal anti-SAMHD1 (Proteintech, 12586-1-AP) for 30 min at 4°C. We previously reported that the levels of SAMHD1 detected by flow cytometry correlated with western blot analysis [35]. Gag stainings were performed with the KC57 mAb (Beckman and Coulter) or with anti-capsid mAb clones 183-H12-5C (NIH AIDS Reagent Program) and 25-26A (Hybridolab, Institut Pasteur). MxA staining was performed with MN143 mAb (provided by Otto Haller) in PBS-Saponine 0.05% for 30 min at 4°C [70]. Samples were analyzed with a FACS CANTO II (Becton Dickinson).

### HIV-2 binding assay

HIV-2 GL-AN and ROD-GFP pseudotyped or not with the VSV-G envelope (50 and 150 ng mL^−1^ of p27) were used to infect 3 × 10^5^ MDDCs in 300 μL at 4°C, in presence of 10 mM Hepes. After 2 h, cells were extensively washed in PBS. Two third of the cells were resuspended in 200 μL PBS + 20 μL Lysis Buffer from the SIV p27 ELISA kit (Zeptometrix). The amount of cell-associated p27 was measured by ELISA.

### HIV-2 fusion assay

Viral fusion was assessed as described [54,55]. Briefly, ultracentrifuged HIV-2 virions containing the Blam-Vpr2 fusion protein (a gift of Matthias Dittmar, [55]) were used to infect 1.5 × 10^5^ MDDCs in 100 μL at 37°C, in presence of 10 mM Hepes and 2 mg.mL-1 DEAE Dextran (Sigma). After 2 hours, cells were washed in cold CO2-independent medium (Invitrogen), without FBS, resuspended in cold CO2-independent medium supplemented with 10% FBS and incubated with the CCF2-AM substrate (CCF2-AM kit, Invitrogen), in the presence of 1.8 mM Probenecid (Sigma), for 2 hours at room temperature. Cells were extensively washed in cold CO2-independent medium and fixed in PBS-PFA 4% for 5 min. The cleaved CCF2-AM fluorescence (excitation at 405 nm, emission at 450 nm) was then immediately measured by flow cytometry using the DAPI channel, on a FacsCanto II (BD).

### HIV-2 DNA quantification

Infected cells DNA was extracted using the DNeasy Blood and Tissue kit (Qiagen). 50 ng of total DNA were used with the Platinum qPCR SuperMix-UDG (Life Technologies) and amplified on an Eppendorf Mastercycler ep realplex^2^ apparatus. HIV-2 Gag primers and probe, as well as PCR conditions have been described [71].

### Statistical analysis

Statistic analysis was performed with the Mann–Whitney test.

## Additional file

Additional file 1: Figure S1. Flow cytometry analysis of CD4+ T cells and MDDCs. a. Staining of resting and activated CD4+ T cells. Unstimulated CD4+ T cells and PHA-activated cells (day 3 post activation) were stained for CD69 and Ki67 (activation and proliferation markers, respectively) and analysed by flow cytometry. The FSC/SSC gatings are depicted. The isotype control is in grey. One representative donor is shown. b. MxA staining of MDDCs. MDDCs were exposed to HIV-2 GL-AN WT (VSV) (50 ng p27 mL-1), with or without AZT, as described in Figure 3. After 3 days, the levels of MxA were measured by flow cytometry. One representative experiment is depicted. c. HIV-2 Gag levels in MDDCs at day 4 post infection. MDDCs were exposed to HIV-2 ROD, GL-AN (150 ng p27 mL-1) and GL-AN WT (VSV) and GL-AN ∆Vpx (VSV) (50 ng p27 mL-1). After 4 days, the levels of Gag and SAMHD1 were measured by flow cytometry. Data are Mean of 2 independent donors.
