# Supplementary figures and images for: HIV-2 infects resting CD4+ T cells but not monocyte-derived dendritic cells
Source: Retrovirology. 2015 Jan 13;12:2. doi: 10.1186/s12977-014-0131-7 (PMC4307230; doi:10.1186/s12977-014-0131-7)

Fig S1.

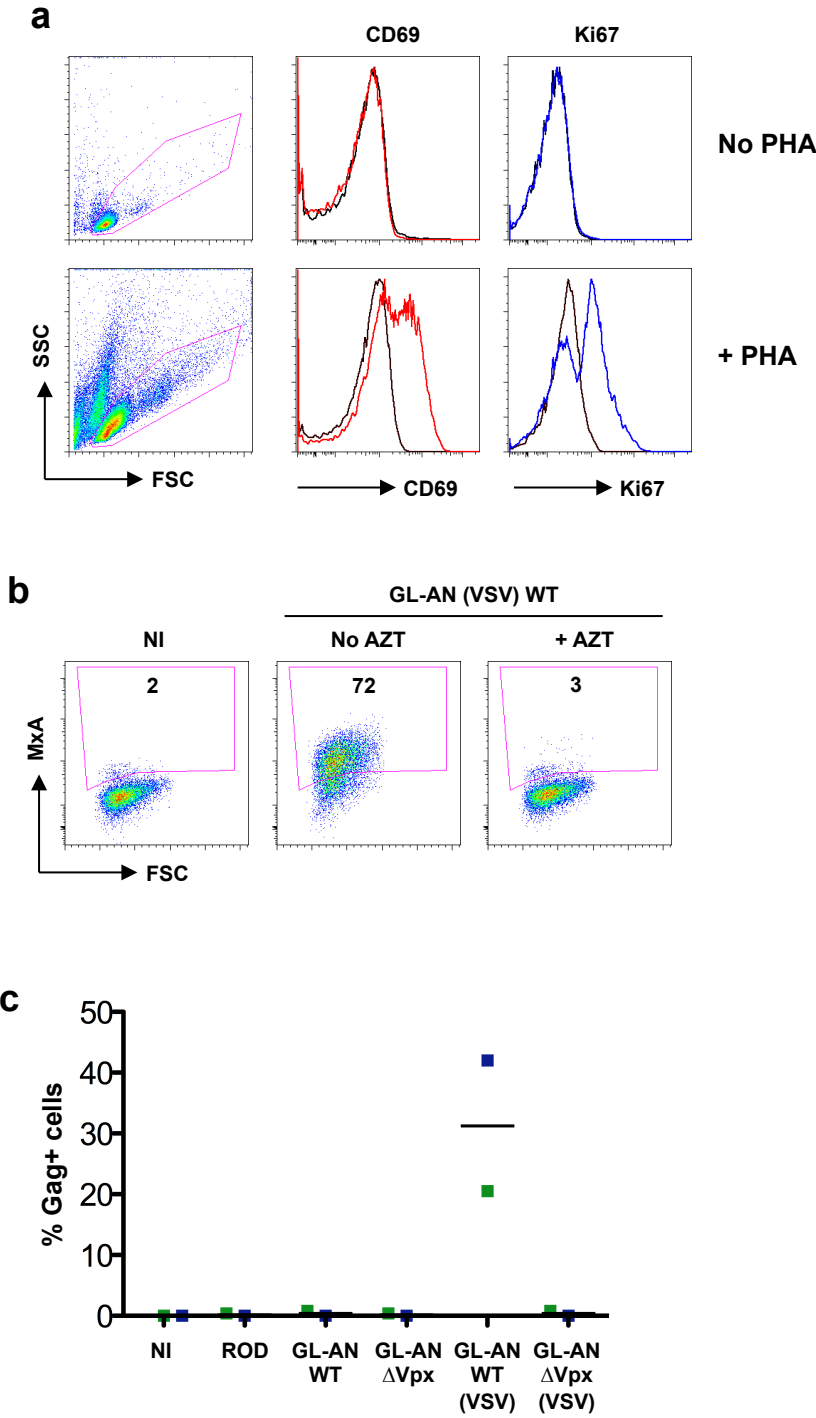

Supplement: Additional file 1: Figure S1. — Flow cytometry analysis of CD4+ T cells and MDDCs. a. Staining of resting and activated CD4+ T cells. Unstimulated CD4+ T cells and PHA-activated cells (day 3 post activation) were stained for CD69 and Ki67 (activation and proliferation markers, respectively) and analysed by flow cytometry. The FSC/SSC gatings are depicted. The isotype control is in grey. One representative donor is shown. b. MxA staining of MDDCs. MDDCs were exposed to HIV-2 GL-AN WT (VSV) (50 ng p27 mL-1), with or without AZT, as described in Figure 3. After 3 days, the levels of MxA were measured by flow cytometry. One representative experiment is depicted. c. HIV-2 Gag levels in MDDCs at day 4 post infection. MDDCs were exposed to HIV-2 ROD, GL-AN (150 ng p27 mL-1) and GL-AN WT (VSV) and GL-AN ∆Vpx (VSV) (50 ng p27 mL-1). After 4 days, the levels of Gag and SAMHD1 were measured by flow cytometry. Data are Mean of 2 independent donors. [file 12977_2014_131_MOESM1_ESM.pdf]
